# Nasal-type NK/T cell lymphoma: clinical features and treatment outcome

**DOI:** 10.1038/sj.bjc.6602502

**Published:** 2005-03-29

**Authors:** J Lee, W S Kim, Y H Park, S H Park, K W Park, J H Kang, S S Lee, S I Lee, S-H Lee, K Kim, C W Jung, Y C Ahn, Y H Ko, K Park

**Affiliations:** 1 Division of Hematology-Oncology, Department of Medicine, Samsung Medical Center, Sungkyunkwan University School of Medicine, 50 Ilwon-dong Kangnam-ku, Seoul 135-710, Korea; 2 Department of Hematology-Oncology, Korea Cancer Center Hospital, Seoul, Korea; 3 Department of Hematology-Oncology, Gachun Medical School Gil Medical Center, Incheon, Korea; 4 Department of Pathology, Korea Cancer Center Hospital, Seoul, Korea; 5 Department of Hematology-Oncology, Dankook University School of Medicine, Seoul, Korea; 6 Department of Radiation Oncology, Samsung Medical Center, Sungkyunkwan University School of Medicine, Seoul, Korea; 7 Department of Pathology, Sungkyunkwan University School of Medicine, Seoul, Korea

**Keywords:** extra-nasal, natural killer cell lymphoma, treatment

## Abstract

Nasal-type NK/T cell lymphoma is an increasingly recognised disease entity of aggressive clinical behaviour. The objective of this study was to investigate clinical features and treatment outcomes in patients with nasal-type NK/T cell lymphoma. From January 1991 to December 2003, 26 patients diagnosed as nasal-type NK/T cell lymphoma were included in the analysis. One half of patients presented with poor performance status (ECOG ⩾2); 46% of patients were categorised as high intermediate or high-risk group according to IPI; and 46% of patients were diagnosed at advanced stage. The median survival for 26 patients with nasal-type NK/T cell lymphoma was 7.4 months (95% CI, 0.1, 16.9). The treatment outcome of primary anthracycline-based chemotherapy was poor: 60% CR rate in localised disease and 0% CR rate in advanced disease. After a median follow-up of 24.4 months (range 3.1–99.0) in patients with localised disease who had achieved a CR (range 29.6–165.7), three patients (50.0%) developed disease recurrence at 6.1, 21.8, and 52.1 months, respectively, and all patients presented with locoregional failure. The predictive factors for poor survival were of age greater than 60, advanced stage and poor performance in multivariate analysis. In conclusion, Nasal-type NK/T cell lymphomas showed a poor response to the conventional anthracycline-based chemotherapy, and thus an investigation for an innovative therapy is urgently needed to improve survival in these patients.

Extranodal NK/T cell lymphoma is subcategorised into nasal and nasal-type NK/T cell lymphomas according to the major sites of anatomic involvement (Jaffe *et al*, 1996, 1999). The nasal NK/T cell lymphoma commonly presents with midline facial destructive disease, shows a strong association with Epstein–Barr virus (EBV), and occurs prototypically within the nasal cavity (Liang *et al*, 1995; Jaffe, 1996; Jaffe *et al*, 1996; Hirakawa *et al*, 1999; Cheung *et al*, 2002). It was previously categorised as angiocentric lymphoma in the Revised European-American Lymphoma (REAL) classification, and has been recently recategorised as extranodal NK/T cell lymphoma in the new WHO classification of lymphoid neoplasms (Harris *et al*, 1994; Jaffe, 1996; Jaffe *et al*, 1996; Harris *et al*, 1999).

In limited cases, NK/T cell lymphoma may predominantly occur in extranasal sites without involvement of nasal cavity or nasopharynx. It is not until recently that this form of extranodal lymphoma has been recognised to present with highly aggressive clinical course and share similar immunophenotypic profile with nasal NK/T cell lymphoma (Kern *et al*, 1992; Wong *et al*, 1992). This disease entity was recognised as an independent form of disease and defined as nasal-type NK/T cell lymphoma in Hong Kong workshop in 1994 (Jaffe, 1996). The largest series on nasal-type NK/T cell lymphoma reported a retrospective review of 34 cases and observed that these patients were often refractory to combined chemotherapy (Chan *et al*, 1997). As most published reports on NK/T cell lymphomas mainly focus on the clinicopathologic features, the role of chemotherapy on the clinical outcome as well the optimal treatment approach needs to be defined. None of the previous studies analysed the treatment outcome of primary chemotherapy in nasal-type NK/T cell lymphoma.

As nasal-type NK/T cell lymphoma is known to be one of the most aggressive lymphomas, it is imperative to offer an appropriately aggressive treatment at an early stage of disease. We report the clinical features and treatment outcome of nasal-type NK/T cell lymphoma patients, especially after anthracycline-based chemotherapy.

## PATIENTS AND METHODS

In total, 26 patients, who were diagnosed as nasal-type NK/T cell lymphoma between January 1991 to December 2003, were included in the analysis. All patients were histologically confirmed of NK/T cell lymphoma, according to WHO classification and had proven NK/T cell type by immunohistochemistry, flow cytometry, or EBV *in situ* hybridisation. Nasal NK/T cell lymphoma, blastic NK-cell leukaemia, aggressive NK cell lymphoma/leukaemia, and peripheral T cell lymphoma, unspecified were excluded from the analysis. Nasal NK/T cell lymphoma was defined as lymphomas occurring within nasal cavity and/or upper aerodigestive tract such as oral cavity, palate, larynx, pharynx, and tonsil. Nasal-type NK/T cell lymphoma was defined as those primarily involving outside nasal cavity/nasopharynx or upper aerodigestive tract. Patients with lesions within the nasal cavity and secondary spread to other organs were categorised as nasal NK/T cell lymphoma and were excluded from the analysis. The pathologic diagnosis of nasal-type NK/T cell lymphoma was based on the following criteria: expression of cytoplasmic CD3 and CD56 and positivity for EBV *in situ* hybridisation. If EBV *in situ* hybridisation was negative, the immunophenotype studies should demonstrate cytoplasmic CD3 expression and positive cytotoxic molecules such as TIA-1.

The following clinical data were collected from the record: patient demographics, complete blood count, lactic dehydrogenase (LDH) level, Ann Arbor stage, IPI, bone marrow findings, the presence of B symptoms, performance status, date of diagnosis, type of treatment, treatment response, date of relapse, date of last follow-up, live status, and cause of death.

### Histology

In all cases, haematoxylin–eosin-stained slides were reviewed by two pathologists. Immunophenotyping was performed using a panel of monoclonal antibodies including antibodies against cytoplasmic CD3 (Dakopatts, Copenhagen, Denmark), CD20 (Dakopatts), CD56 (Monosan, Uden, the Netherlands), and TIA-1 (Coulter, Hialeah, FL, USA). EBV RNA was detected by an *in situ* hybridisation technique. Briefly, paraffin sections were pretreated with xylene, followed by treatment with proteinases K and hybridised with FITC-conjugated EBV oligonuecleotides (Dakopatts) complementary to the nuclear RNA portion of the EBER 1 and 2 genes.

### Treatment

The anthracycline-based chemotherapy regimen used were as following: CHOP (cyclophosphamide, doxorubicin, vincristine, prednisolone), dose-escalated CHOP(deCHOP), COPBLAM (cyclophosphamide, vincristine, prednisone, bleomycin, doxorubicin, procarbazine), and EPOCH (etoposide, doxorubicin, vincristine, cyclophosphamide, prednisolone). The nonanthracycline-based chemotherapy regimens were DICE (dexamethasone, ifosfamide, cisplatin, etoposide) and CVP (cyclophosphamide, vincristine, prednisone). CHOP regimen consisted of cyclophosphamide(750 mg m^−2^ given intravenously on day 1), doxorubicin (50 mg m^−2^ given intravenously on day 1), vincristine (1.4 mg m^−2^ but not more than 2.0 mg given intravenously on day 1), and prednisolone (100 mg daily, given orally on days 1–5). Dose-escalated CHOP consisted of cyclophosphamide(1250 mg m^−2^ given intravenously on day 1), doxorubicin (75 mg m^−2^ given intravenously on day 1), vincristine (1.4 mg m^−2^ but not more than 2.0 mg given intravenously on day 1), and prednisolone (100 mg daily, given orally on days 1–5) with G-CSF support until neutrophil count recovery. The chemotherapy cycles were repeated at 21-day intervals. The alternative regimens were COPBLAM (cyclophosphamide 400 mg m^−2^, vincristine 1.0 mg m^−2^, and doxorubicin 50 mg m^−2^ intravenously on day 1, bleomycin 15 mg intravenously on day 14, procarbazine 100 mg m^−2^ and prednisolone 40 mg m^−2^ daily, given orally on days 1±10); and EPOCH (etoposide 50 mg m^−2^, vincristine 0.4 mg m^−2^, and doxorubicin 10 mg m^−2^, all given in a continuous infusion on days 1–4 with cyclophosphamide 750 mg m^−2^ i.v. bolus on day 6 and oral prednisone 60 mg m^−2^ on days 1–6). DICE regimen consisted of dexamethasone 10 mg q.i.d., ifosfamide 1 g m^−2^, cisplatin 25 mg m^−2^, etoposide 100 mg m^−2^, and mesna uroprotection daily × 4 given every 28 days, and CVP was cyclophosphamide 1000 mg m^−2^ i.v. on day 1, vincristine 1.4 mg m^−2^ (max. 2 mg) i.v. on day 1, and prednisolone 100 mg daily, given orally on days 1–5.

In patients with localised disease, involved-field radiotherapy (IFRT) was given at physician's discretion following chemotherapy. IFRT began 3 weeks after the completion of planned chemotherapy. The total radiotherapy dose was 45 Gy administered over 5 weeks by conventional fractionation schedule (1.8 Gy/fraction, 5 fractions/week) to the prechemotherapy gross disease extent.

### Response criteria

The treatment response was assessed according to standard response criteria (Cheson *et al*, 1999). Complete remission (CR) was defined as no evidence of residual disease; partial response (PR) was at least a 50% reduction in the tumour burden from the onset of treatment; no response was less than 50% reduction in tumour burden or disease progression. CR/unconfirmed (Cru) was defined as the reduction of mass by more than 75% with no newly developed lesions. Assessment of response was evaluated by follow-up clinical, radiologic, and laboratory studies as determined relevant by the clinician.

### Statistical analysis

Survival rates were compared for statistical differences by using log-rank analysis. *P*-values less than 0.05 were considered statistically significant and all *P*-values correspond to two-sided significance tests. Overall survival (OS) and disease-free survival (DFS) were estimated using the Kaplan–Meier product-limit method. Disease-free survival was calculated from the date treatment began to the date when the disease progression was recognised or the date of the last follow-up visit. Overall survival was measured from the date of diagnosis to the date of death or the last follow-up visit.

## RESULTS

### Clinical characteristics and OS

**Figure 1:**
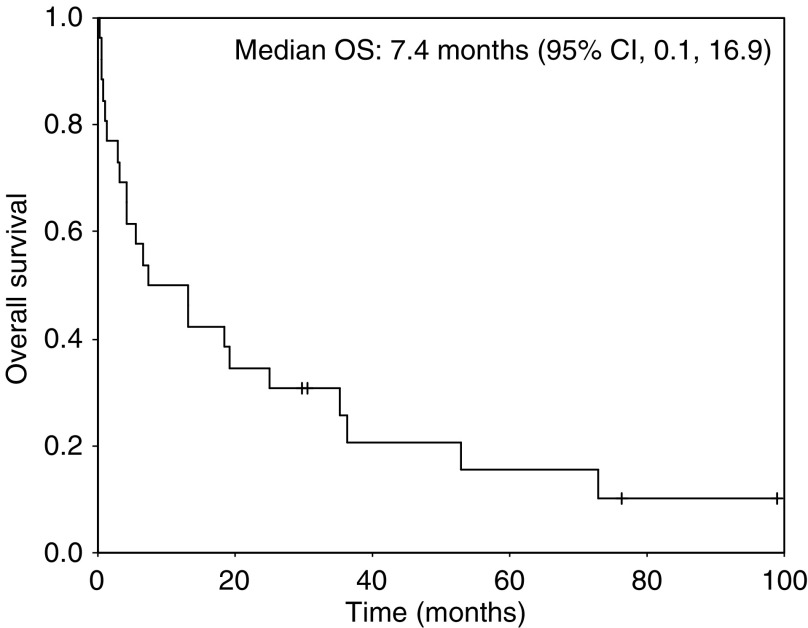
Overall survival according to subtypes.

The clinical characteristics of patients with nasal-type NK/T cell lymphoma are listed in Table 1. The median age at diagnosis was 50 years (range, 22–81 years). There was a male preponderance with a male to female ratio of 2.3 : 1. One half of patients presented with poor performance status (ECOG ⩾2), 46% of patients were categorised as high intermediate or high-risk group according to IPI, and 46% of patients were diagnosed at advanced stage. B symptoms occurred in 11 (42.3%) patients. An OS curve is shown in Figure 1 for all patients (*n*=26). The median survival for patients with nasal-type NK/T cell lymphoma was 7.4 months (95% CI, 0.1, 16.9). The median follow-up duration for living patients was 53.4 months with a range of 29.6–99.0 months. The major sites of involvement were as following: gastrointestinal tract (*n*=8), cutaneous (*n*=7), soft tissues (*n*=8), pleura (*n*=1), and liver (*n*=2) (Table 2).

### Localised nasal-type NK/T cell lymphoma

Of 14 patients with early stage nasal-type NK/T cell lymphoma (stage I and II), 10 patients received primary anthracycline-based chemotherapy (nine CHOP, one COPBLAM). Two patients underwent primary surgical resection due to their initial presentation of bowel obstruction and received CHOP chemotherapy as postoperative treatment. One patient presented with soft tissue mass at calf and achieved CR following mass excision and IFRT but experienced local relapse. One remaining patient had best supportive care due to old age and patient's refusal for further treatment. Of 10 patients who received anthracycline-based chemotherapy with or without IFRT as a primary treatment, six patients (60.0%) achieved CR. After a median follow-up of 24.4 months of these patients (range 3.1–99.0), three patients (50.0%) developed disease recurrence at 6.1, 21.8, and 52.1 months, respectively. The patterns of recurrence were locoregional in all three patients. Three patients, who achieved CR after CHOP chemotherapy followed by IFRT, remain alive in remission at 29.6, 30.5, and 99.0 months, respectively. Of five patients who were refractory to the initial anthracycline-based chemotherapy, two patients died after two and six cycles of CHOP chemotherapy, respectively, and three patients received salvage treatment with either high-dose cytarabine or ifosfamide-based chemotherapy. All of the five anthracycline-refractory patients died between 3.1 and 72.8 months.

### Disseminated nasal-type NK/T cell lymphoma

In total, 12 patients (46.2%) presented with disseminated disease and seven patients received primary anthracycline-based chemotherapy (six CHOP, one EPOCH). All seven patients developed progressive disease despite of chemotherapy and died of disease. Of these seven patients, only two patients (28.6%) have tolerated more than two cycles of chemotherapy, while four patients (57.1%) died after one cycle of chemotherapy. Of the remaining five patients, two patients received nonanthracycline based chemotherapy (DICE and CVP) and three patients did not receive chemotherapy. Of 12 patients with disseminated disease, six patients (85.7%) died of disease progression or treatment related complications within less than 7 months from the date of diagnosis.

### Clinical predictors of survival

**Figure 2:**
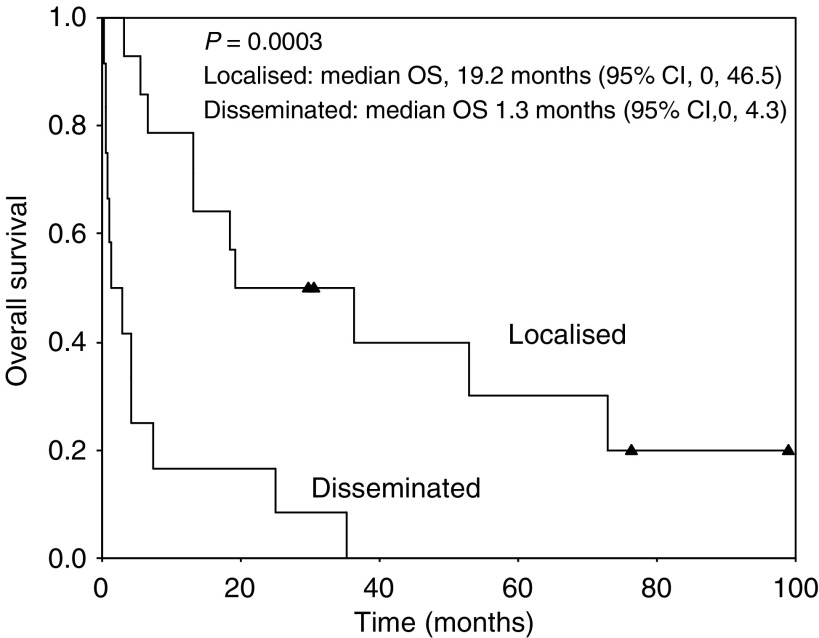
Overall survival curve according to stage.

The important clinical factors predicting reduced survival were age greater than 60, poor performance, presence of B symptoms, high IPI score, advanced stage, and bone marrow involvement at univariate level (Table 3, Figure 2). In stepwise Cox multivariate regression analysis, age greater than 60, advanced stage, and poor performance had an independent influence on poor survival.

## DISCUSSION

As nasal-type NK/T cell lymphoma has recently been recognised as an independent disease entity, neither the clinical features nor the optimal therapeutic approach has been defined yet. This report is the first paper to describe clinical behaviour and the treatment outcome exclusively in patients with nasal-type NK/T cell lymphoma. We analysed the treatment outcome of anthracycline-based chemotherapy (CHOP in 15 out of 17 patients, 88.2%) in this rare subtype of lymphoma.

Nasal-type NK/T cell lymphoma has been introduced at the Hong Kong workshop held in 1994 (Jaffe *et al*, 1996). This disease entity encompasses all NK/T cell lymphomas occurring outside nasal cavity and nasopharynx and is notorious for its aggressive clinical course. The definition of nasal-type is confusing, which resulted in different categorisation by various institutions (Kern *et al*, 1992; Wong *et al*, 1992; Peiper, 1993; Nakamura *et al*, 1995; Jaffe *et al*, 1996, 1999; Kobashi *et al*, 1996; Chan *et al*, 1997; Bekkenk *et al*, 2004; Ko *et al*, 2004). We defined nasal-type lymphoma as lesions primarily involving skin, soft tissues, visceral organs such as liver, spleen, and gastrointestinal tract without any lesions within the nasal cavity and/or upper aerodigestive tract such as oral cavity, tonsil, pharynx, and larynx.

Several studies have reviewed small series of patients and suggested an early administration of highly aggressive treatment for nasal-type NK/T cell lymphoma. Chan *et al* (1997) summarised 32 cases of nasal-type NK/T cell lymphoma and described the aggressive clinical course of the disease. Although survival data or detailed treatment outcome were not provided, they found the disease to be refractory to combination chemotherapy since 24 patients died in 1 week to 3 years, with a median of 3.5 months despite of chemotherapy. Mraz-Gernhard *et al* (2001) reported 30 cases of nasal-type NK/T cell lymphomas with cutaneous presentation and primary anthracycline-based chemotherapy was administered in 10 patients, where five patients achieved CR with short duration of response ranging from 1.5 to 4 months. A small retrospective review on the treatment outcome of the first-line anthracycline-based chemotherapy in extranodal NK/T cell lymphoma reported a low CR rate of 28.6% in 14 cases of nasal-type NK/T cell lymphoma patients (Kim *et al*, 2003). The Dutch group recently analysed 38 nasal-type NK/T cell lymphoma patients from the literature, of whom 70% received CHOP-like chemotherapy and showed a median survival of 6 months (Bekkenk *et al*, 2004). This group concluded that doxorubicin-based chemotherapy was insufficient for effective disease control for both nasal and nasal-type NK/T cell lymphomas.

Likewise, the median survival of 26 nasal-type NK/T cell lymphoma patients was only 7.4 months (95% CI, 0.1, 16.9) in our series. The overall response to the primary anthracycline-based chemotherapy in 17 patients was poor (overall CR rate 35.3%, 95% C.I. 12.6, 58.0). We have previously reported that three or four cycles of CHOP followed by IFRT was not satisfactory for treating patients with localised nasal NK/T cell lymphoma due to low CR rate (six of 15 patients, 40%, Kim *et al*, 2001). Owing to the limitations of a retrospective study and the small number of patients in the series, conclusions on the optimal treatment or pattern of failure cannot be made. In localised disease, all three relapsed patients showed local failure while two live patients in remission had received IFRT following four cycles of CHOP. This may suggest a possible role of IFRT for local disease control in nasal-type NK/T cell lymphoma, although IFRT alone has shown to be unsatisfactory due to frequent systemic failures (Kim *et al*, 2000).

One possible explanation for the resistance to the standard chemotherapy would be a high expression of multidrug resistance (P-glycoprotein positive) phenotype in NK/T cell lymphoma patients. One study reported that an immunohistochemical staining against MDR in 28 NK/T cell lymphoma patients with primary cutaneous involvement revealed 78.6% MDR positivity, although its clinical correlation is yet to be defined (Mraz-Gernhard *et al*, 2001).

Considering the poor outcome of standard treatments, few groups designed an innovative therapy. The Chinese group reported a successful treatment in a patient with advanced nasal-type NK/T cell lymphoma with four courses of l-asparaginase combined with vincristine and prednisone as an induction therapy followed by six courses of CHOP as a consolidation treatment (Obama *et al*, 2003). Lee *et al* (2004) recently administered a combination chemotherapy of ifosfamide, methotrexate and etoposide in six extranasal NK/T cell lymphoma patients and reported a 20% CR rate.

In conclusion, we suggest that the anthracycline-based chemotherapy should not be used as a primary treatment for localised or advanced nasal-type NK/T cell lymphoma. Furthermore, because of the tendency of local failure for localised disease, the addition of radiation therapy to chemotherapy should be incorporated in a prospective trial. Nasal-type, NK/T cell lymphoma is a highly aggressive disease and the exploration for highly active drug is crucial to improve survival.

## Figures and Tables

**Table 1:** Patient characteristics

	**Patients**	
	**No.**	**%**
Median age (years), range	50 (22–81)	
Sex (M : F)	18 : 8	
Performance status (ECOG⩾2)	13	50.0
B symptom (+)	11	42.3
Stage I, II	14	53.9
		
*IPI*		
Low	7	26.9
Low intermediate	7	26.9
High intermediate	6	23.1
High	6	23.1
		
LDH (high)	16	61.5
EBV ISH (+)	18/23	78.3
BM involvement	2	7.7

**Table 2:** Treatment outcome

**No.**	**Major site**	**Stage**	**Initial treatment**	**Treatment response**	**Status**	**F/U (months)**	**Pattern of failure/remark**
1	Colon	Localised	CHOP	PR	DOD	6.7	
2	Terminal ileum	Localised	Surgery → CHOP	PD	DOD	3.1	
3	Colon	Disseminated	Surgery	N/A	DOD	0.9	
4	Duodenum	Disseminated	CHOP	PD	DOD	35.4	
5	Ileum	Disseminated	EPOCH	PD	DOD	0.6	
6	Ileum	Localised	Surgery → CHOP	CR	DOO	52.9	
7	Stomach, spleen	Disseminated	CHOP	PD	DOD	1.3	
8	Skin, leg	Disseminated	Supportive care	N/A	DOD	0.4	
9	Skin, foot	Localised	deCHOP → RT	CR	AIR	30.5	No relapse
10	Skin, elbow	Disseminated	Supportive care	N/A	DOD	24.9	
11	Skin, eyelid	Disseminated	CVP	PD	DOD	0.3	
12	Skin, eyelid	Localised	COPBLAM	PD	DOD	72.8	
13	Skin, eyelid	Localised	DeCHOP → RT	CR	AIR	29.6	No relapse
14	Skin, hand	Localised	CHOP	CR	DOD	18.5	Systemic relapse
15	Soft tissue, inguinal LN	Localised	CHOP → RT	CR	DOD	36.4	Locoregional relapse → salvage chemo → DOD
16	Jejunum, cervical LNs	Disseminated	CHOP	PD	DOD	7.4	
17	Liver, porta hepatic LNs	Disseminated	CHOP	PD	DOD	2.8	
18	Muscle, submental LN	Localised	CHOP	PD	DOD	5.6	
19	Soft tissue, calf	Localised	RT	CR	AWD	76.3	Locoregional relapse → RT → PD → salvage chemo → AWD
20	Muscle, masseter	Localised	CHOP	PD	DOD	13.2	
21	Soft tissue, elbow	Localised	CHOP → RT	CR	AIR	99.0	No relapse
22	Soft tissue, thigh	Localised	Supportive care	N/A	DOD	13.0	
23	Soft tissue, arm	Disseminated	DICE	PD	DOD	4.3	
24	Soft tissue, hand	Localised	CHOP	CR	DOD	19.2	Locoregional relapse → salvage chemo → DOD
25	Liver, spleen	Disseminated	CHOP	PD	DOD	4.2	
26	Pleura, lung	Disseminated	CHOP	PD	DOD	1.1	

DOD=dead of disease; DOO=dead of other cause; AIR=alive in remission; AWD=alive with disease; CHOP=cyclophosphamide, doxorubicin, vincristine, prednisolone; dose-escalated CHOP=deCHOP; COPBLAM=cyclophosphamide, vincristine, prednisone, bleomycin, doxorubicin, procarbazine; EPOCH=etoposide, doxorubicin, vincristine, cyclophosphamide, prednisolone; DICE=dexamethasone, ifosfamide, cisplatin, etoposide; CVP=cyclophosphamide, vincristine, prednisone.

**Table 3:** Prognostic factors

**Variables**	**OS (*P*-value)**	
	**Univariate analysis**	**Multivariate analysis**
Age (>60)	0.0283	0.0261
Sex	0.7947	0.4681
PS (ECOG 2–4)	0.0004	0.0031
B symptom	0.0202	0.6352
LDH	0.0822	0.3851
IPI	0.0018	0.2692
BM (+)	0.0161	0.2121
EBV (+)	0.7008	0.7310
Stage (disseminated)	0.0003	0.0017
